# Is beta-carotene consumption associated with thyroid hormone levels?

**DOI:** 10.3389/fendo.2023.1089315

**Published:** 2023-05-26

**Authors:** Bahareh Farasati Far, Nima Broomand Lomer, Hossein Gharedaghi, Hadi Sahrai, Golnaz Mahmoudvand, Arian Karimi Rouzbahani

**Affiliations:** ^1^ Department of Chemistry, Iran University of Science and Technology, Tehran, Iran; ^2^ Faculty of Medicine, Guilan University of Medical Sciences, Guilan, Iran; ^3^ School of Medicine, Zanjan University of Medical Sciences, Zanjan, Iran; ^4^ Student Research Committee, Tabriz University of Medical Sciences, Tabriz, Iran; ^5^ Student Research Committee, Lorestan University of Medical Sciences, Khorramabad, Iran; ^6^ USERN Office, Lorestan University of Medical Sciences, Khorramabad, Iran

**Keywords:** beta carotene, thyroid hormone levels, thyroid cancer, hormonal imbalance, vitamin A, retinol

## Abstract

The thyroid hormones play a pivotal role in various physiological processes, including growth, metabolism regulation, and reproduction. While non-modifiable factors are known to impact thyroid function, such as genetics and age, nutritional factors are also important. Diets rich in selenium and iodine are conventionally acknowledged to be beneficial for the production and release of thyroid hormones. Recent studies have suggested a potential link between beta-carotene, a precursor to vitamin A (retinol), and thyroid function. Beta-carotene is known for its antioxidant properties and has been shown to play a role in the prevention of various clinical conditions such as cancer and cardiovascular and neurological diseases. However, its impact on thyroid function is still unclear. Some studies have suggested a positive association between beta-carotene levels and thyroid function, while others have found no significant effect. Conversely, the hormone produced by the thyroid gland, thyroxine, enhances the conversion of beta-carotene to retinol. Furthermore, vitamin A derivatives are being explored as potential therapeutic options for thyroid malignancies. In this review, we highlight the mechanisms through which beta-carotene/retinol and thyroid hormones interact and review the findings of clinical studies examining the association between beta-carotene consumption and thyroid hormone levels. Our review underscores the need for further research to clarify the relationship between beta-carotene and thyroid function.

## 1 Introduction

Thyroid hormones (THs) have various functions in almost all cell types. Thyroid malfunction is prevalent across the globe, and it is identified as a controllable and treatable disease. Taking into account the greater knowledge of thyroid disorders we do have today and the accessibility of effective diagnostic tests for assessing THs, it is vital to manage TH secretion abnormalities cautiously (1). Subclinical hypothyroidism is when thyroid-stimulating hormone (TSH) levels are slightly elevated (between 4.6 and 8.0 mIU/ml) despite normal free T4 levels, while overt hypothyroidism is characterized by decreased free T4 levels. On the other hand, subclinical hyperthyroidism is marked by mildly suppressed TSH levels (usually still above 0.1 mIU/ml) and normal TH levels, while overt hyperthyroidism is characterized by increased TH levels (2). In most cases, hypothyroidism and hyperthyroidism are caused by pathological processes within the thyroid gland known as primary thyroid disease. However, in uncommon situations, hypothyroidism and hyperthyroidism are caused by dysfunction of the hypothalamus, pituitary, struma ovary, or responsive thyroid cancer metastases (3, 4). Autoimmunity is the most common cause of thyroid gland dysfunction in iodine-deficient populations, resulting in Graves’ disease, Hashimoto’s thyroiditis, and pregnancy hypothyroidism. Also, recent studies reported that oxidative stress (OS) is associated with hyperthyroidism and hypothyroidism (5). The mechanism of these two clinical conditions is different, and OS is produced by separate pathways: elevated reactive oxygen species (ROS) formation in hyperthyroidism and decreased antioxidant availability in hypothyroidism (6). In addition, THs can play an oxidative role in target cells (7).

Beta-carotene (β-carotene), known as a fat-soluble pigment, is found in red, orange, and yellow vegetables and fruits. When the body is deprived of vitamin A, beta-carotene is transformed into vitamin A. β-Carotene is an antioxidant and a substance that prevents activated oxygen molecules from damaging cells (8). Phytochemicals like β-carotene are crucial in fighting free radicals as they target various enzymes across multiple pathways (9). Also, the intake of other antioxidants such as vitamin C and vitamin E in rats showed that they can reduce oxidative stress (10, 11). Alongside β-carotene, selenium is also an antioxidant that is associated with TH levels. Rostami et al. reported that decreased selenium level in the serum is a trigger of oxidative stress, resulting in thyroid dysfunction (12). As a result, these findings can be helpful in the treatment of thyroid disorders and in decreasing harmful events caused by OS. However, there is a lack of both *in vivo* and *in vitro* studies to understand the association and mechanism between β-carotene and TH levels with OS events and other thyroid-related complications. This study was conducted in order to review the mechanisms through which beta-carotene/retinol and THs interact and further review the findings of clinical studies in this regard.

The aim of this review paper is to investigate the potential association between beta-carotene consumption and thyroid hormone levels. Beta-carotene is a precursor to vitamin A, which plays an important role in thyroid hormone synthesis and metabolism. There is growing interest in the potential health benefits of beta-carotene, particularly its ability to prevent chronic diseases such as cancer and cardiovascular disease. However, some studies have suggested that high levels of beta-carotene intake may interfere with thyroid function, leading to alterations in thyroid hormone levels. This review will examine the available evidence on the relationship between beta-carotene consumption and thyroid hormone levels, including both observational and interventional studies. We will also explore the potential mechanisms by which beta-carotene may affect thyroid function and identify areas for further research. Overall, this review aims to provide a comprehensive assessment of the current evidence on the association between beta-carotene consumption and thyroid hormone levels and to inform future research in this area.

Research on the relationship between beta-carotene consumption and thyroid hormone levels is an area of active investigation, and future studies are likely to build on the existing body of research. One possible direction for future research is to conduct clinical trials that examine the effects of beta-carotene supplementation on thyroid hormone levels in humans. This type of study would involve giving participants beta-carotene supplements and monitoring their thyroid hormone levels over time to determine whether there is a causal relationship between beta-carotene consumption and changes in thyroid function. Another potential avenue for future research is to explore the mechanisms by which beta-carotene affects thyroid hormone levels. For example, studies could investigate the role of beta-carotene in regulating the activity of enzymes that are involved in thyroid hormone synthesis or metabolism. In addition, future studies could examine the relationship between beta-carotene consumption and other markers of thyroid function beyond just thyroid hormone levels, such as TSH or thyroid antibodies. This could provide a more comprehensive understanding of the relationship between beta-carotene and thyroid health. Therefore, the field of research on beta-carotene consumption and thyroid hormone levels is still evolving, and future studies are likely to shed more light on this important topic.

## 2 Search strategy

The search strategy used a combination of relevant medical subject headings (MeSH terms) and keywords, including (“beta carotene” OR “beta-Carotene 15,15-Monooxygenase”) AND (“Thyroid Hormones” OR “hypothyroidism” OR “hyperthyroidism” OR “T3 thyroid hormone” OR “T4 thyroid hormones” OR “mechanism of action” OR “thyroid” OR “vitamin A” OR “Retinoid” OR “Retinol” OR “thyroid stimulating hormone” OR “TSH”). The search strategy was designed to be comprehensive and was iteratively refined based on the initial search results. The search results were then screened based on the inclusion and exclusion criteria, and the relevant studies were selected for full-text review.

### 2.1 Inclusion criteria

To identify relevant studies for this review, a comprehensive search strategy was employed using electronic databases, including PubMed, MEDLINE, and Google Scholar. The search was limited to studies published between January 1990 and December 2022, and only English-language articles were included. The following inclusion criteria were used:

- Studies reporting on the relationship between beta-carotene consumption and thyroid hormone levels in humans
- Studies reporting on the effects of beta-carotene supplementation or dietary intake on thyroid function tests, including the levels of TSH, triiodothyronine (T3), and thyroxine (T4)
- Studies that reported beta-carotene intake through dietary sources or supplements
- Studies that included adult human subjects

### 2.2 Exclusion criteria

The following exclusion criteria were used to ensure the relevance and quality of the studies included in this review:

- Studies that did not report on the relationship between beta-carotene consumption and thyroid hormone levels
- Studies that did not include human subjects
- Studies that were not published in English or were published before January 1990 or after December 2022
- Studies that were not peer-reviewed or were of low quality, as assessed by the authors

## 3 Is beta-carotene level different among men and women?

Studies have reported sex differences in beta-carotene levels, with women generally having higher concentrations than men. The underlying mechanisms for these differences are not yet fully understood, but sex hormones such as estrogen and testosterone may play a role. For instance, estrogen can increase the expression of beta-carotene transporter proteins in the liver, leading to higher plasma concentrations of beta-carotene in women (13). Thyroid hormones are important regulators of metabolism, growth, and development in the body. The thyroid gland produces two main hormones: T4 and T3. Several studies have reported sex differences in thyroid hormone levels, with women having higher levels than men. This may be due to the influence of estrogen on thyroid hormone production and metabolism. Estrogen can enhance the conversion of T4 to T3, the more active form of thyroid hormone, leading to higher circulating levels of T3 in women. Additionally, estrogen can increase the number of thyroid hormone receptors in target tissues, further amplifying the hormonal effects (14). The differences in beta-carotene and thyroid hormone levels between men and women may have important implications for overall health and disease risk. For instance, low levels of beta-carotene have been linked to increased risk of cancer, cardiovascular disease, and other chronic conditions. On the other hand, high levels of thyroid hormone may increase the risk of osteoporosis, atrial fibrillation, and other health problems (15). Therefore, understanding the sex differences in these biomarkers can help identify individuals who may be at higher risk of certain diseases and inform targeted prevention and treatment strategies.

## 4 Beta-carotene and retinoid: the peripheral metabolism of thyroid hormones

**Figure 1:**
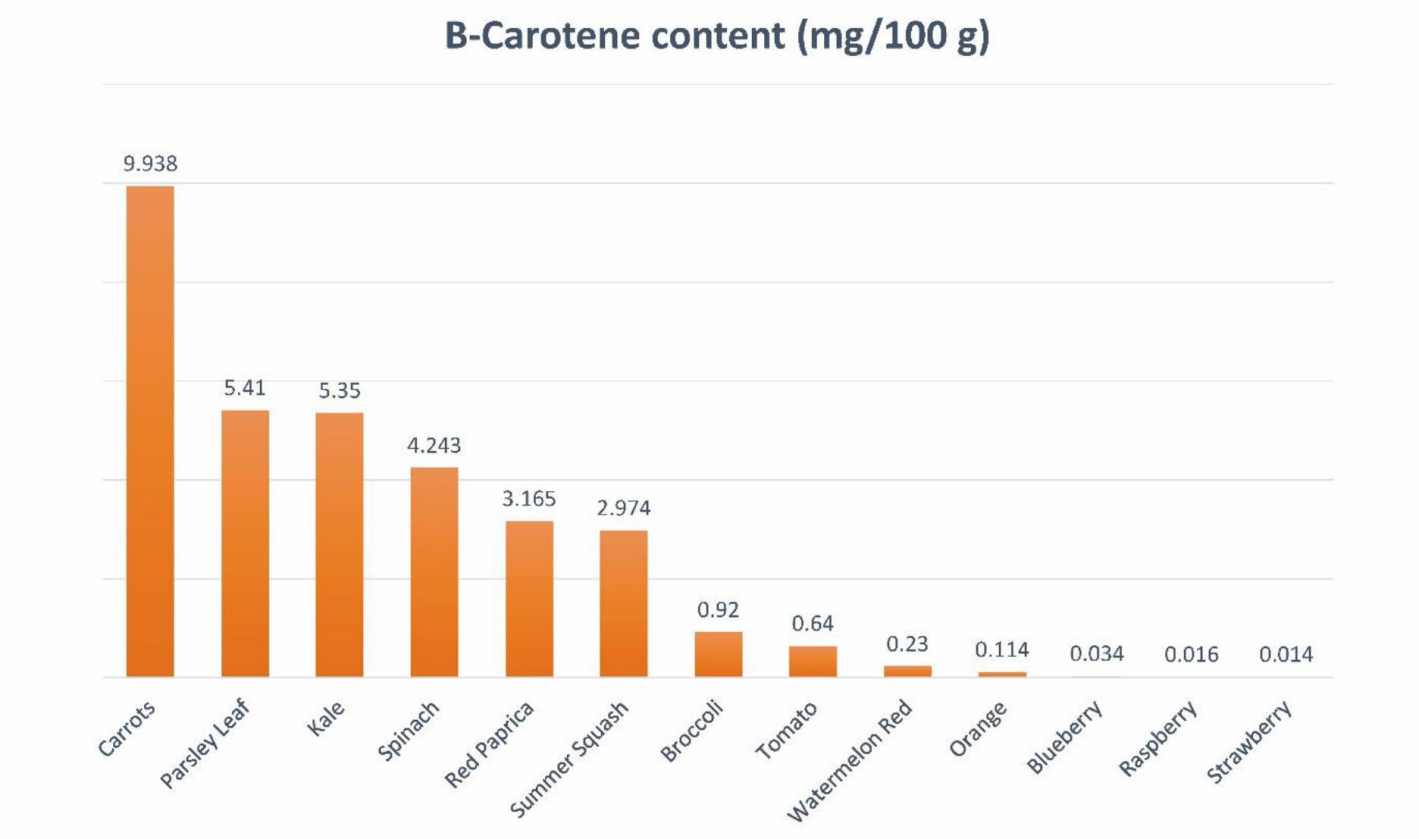
Quantitative sources of β-carotene.

Carotenoids are lipophilic antioxidants copiously present as colored pigments in vegetables and fruits. Carotenoids can be extracted from various parts of plants, including the green parts, flowers, fruits, seeds, roots, and tubers. These compounds are found in a range of vegetables like carrots, pumpkins, spinach, and tomatoes, as well as fruits such as watermelons and raspberries (**Figure 1**). Within plant cells, carotenoids are situated in the organelles of chloroplast known as thylakoid membranes.

There are hundreds of types of carotenoids in nature; however, almost 20 types, including beta-carotene, can be found in human tissues (16). Beta-carotene, a tetra-terpenoid containing two β-ionic rings, is one of the most common carotenoids in the human diet (17). It plays countless biological roles in the human body. The most vital function of beta-carotene is acting as a precursor of vitamin A (8). As beta-carotene cannot be produced in the human body, it must be supplied by consuming plant-based nutrition. In the intestine, beta-carotene is absorbed by enterocytes concurrently with consumed lipids. At the basolateral aspect of the enterocytes, beta-carotene gets cleaved by beta-carotene 15–15′-oxygenase, synthesizing retinal, which then converts to retinoic acid (RA) and retinol (17, 18). Vitamin A derivates subsequently influence physiological growth and development, as well as eyesight, regulation of the immune system, and reproduction (8, 17, 19). In addition to these effects, the endocrine system has a significant link with the retinoid system (20). Thyroid hormones, including T3 and T4, are vital hormones in the metabolism of different organs. These hormones mainly act through the cytosolic functions of T3, which adjust gene expression by attaching to TH receptors (21). RA does not participate in thyroid organogenesis; nevertheless, it plays a key role in preserving an improved thyroid cell phenotype (22). In a flatfish model, it was discovered that dietary vitamin A content significantly affected thyroid follicle development, increasing the number of follicles as well as elevating the colloid content of THs during the pre-metamorphosis stage and reducing THs, particularly T3, in cortical vesicles during the pro-metamorphosis stage (23). Vitamin A contributes to the synthesis of T4 as well as the establishment of an intracellular receptor for T3 (24). It has been observed that vitamin A deficiency damages the formation of thyroglobulin, which is a protein precursor of THs (25, 26). Individuals with elevated serum levels of TSH and decreased or normal free T4 have shown high levels of serum retinol and beta-carotene (27). Patients undergoing RA therapy are supposed to be at an increased risk of hypothyroidism (28). Some may suppose that, as the conversion of beta-carotene to retinol is reduced in patients with hypothyroidism, there must be a negative association between their serum concentrations in these patients. Studies have shown that as retinol synthesized from retinyl esters stacks in the serum, both beta-carotene and retinol levels rise in hypothyroidism (29). Furthermore, certain links have been reported between vitamin A and iodine metabolism. Vitamin A and iodine co-deficiency in children elevates TSH levels and the chance of goiter (30). The use of RA can also decrease the risk of hypothyroidism in patients undergoing radioactive iodine therapy for functional nodules (31, 32). All in all, RA has been linked to thyroid function at different levels of the hypothalamus–pituitary–thyroid axis. **Table 1** represents an overview of the potential therapeutic benefits of vitamin A derivatives in different thyroid disorders.

**Table 1:** Overview of the potential therapeutic benefits of vitamin A derivatives in different thyroid disorders.

Disorder	Therapeutic effects	Ref
Hypothyroidism	Administration of vitamin A increases free T4 concentrations in patients with hypothyroidism.	(33)
Subclinical hypothyroidism	Retinyl palmitate supplementation is beneficial for reducing the risk of subclinical hypothyroidism in women of reproductive age.	(34)
Hyperthyroidism	High doses of vitamin A are effective in decreasing symptoms and metabolic rates in patients suffering from hyperthyroidism.	(35)
Goiter	Vitamin A supplementation in combination with iodized salt reduces the thyroid volume and serum TSH levels significantly.	(36)
Thyroid eye disease	β−Carotene reduces the H_2_O_2_−induced proliferation in eye involvement secondary to thyroid diseases.	(37)
Thyroid cancer	ATRA inhibits the malignant properties of thyroid cancer cells and stimulates apoptosis.	(38)

T4, thyroxine; TSH, thyroid-stimulating hormone; ATRA, all-trans retinoic acid.

## 5 Molecular mechanism of vitamin A metabolization and relation to thyroid hormones

As the understanding of the link between vitamin A and the thyroid grows, many investigators are exploring the molecular structures by which β-carotene and RA affect thyroid performance. RA exerts its effects through nuclear receptors belonging to the steroid family, known as RA receptors (RAR). These receptors, together with the retinoid X receptor (RXR), form heterodimers to bind DNA on RA response element sequences situated in the promoter area to promote or suppress gene expression (20). These interactions may trigger biological pathways involving different tissues and organs (39). The endocrine system has an inseparable relationship with the retinoid system as the RXR provides heterodimers for certain hormone nuclear receptors, including thyroid receptors (20).

A crosstalk between THs and RA signaling has been observed in previous studies. In a preclinical study of mouse models, it was observed that iodothyronine deiodinase type 2 (DIO2) and iodothyronine deiodinase type 3 (DIO3) switching is reduced in fetuses with neural tube defects born from mothers with hyperthyroidism. It should be noted that the enzyme DIO3 degrades T3 to diiodo-l-thyronine and converts T4 to reverse T3, while DIO2 converts T4 to T3. Researchers explained the reduction in DIO3 expression by elevated levels of inhibitory histone in the DIO3 promoter area, indicating that overactive RA signaling can ectopically de-repress TH signaling (40). Fernández et al. evaluated the influence of vitamin A on flatfish development in terms of TH signaling. Gene expression analysis revealed that the mRNA levels of retinoid receptors, retinol-binding protein, and TH receptors including TRαa, TRαb, and TRβ along with TSHβ were upregulated in larvae fed with rotifers with 50 times above normal vitamin A content compared with the control group (23). Froöhlich et al. found that retinol promoted the binding of TSH to thyroid cells but did not influence the expression of the sodium–iodide symporter mRNA and protein (41). Another group of researchers investigated the effects of RA on thyroid function in rats treated with all different doses of RA. The thyroid iodine content diminished in rats treated with all-trans-retinoic-acid (ATRA) for 14 days and rose in those treated with 13-cis-RA. In the ATRA group, sodium–iodide symporter function and thioperoxide activity remained unmodified; however, dual oxidase activity remarkably decreased (42).

RA also regulates the effects of THs on target tissues. It has been reported that ATRA or RAR enhances the uptake of THs through transcriptional upregulation of monocarboxylate transporter in the extraembryonic endoderm development period (43). Furthermore, RAR and thyroid receptors share some cofactors such as cytoplasmic adaptor for RAR and thyroid receptor (CART1). It has been suggested that the C-terminal CoRNR box of CART1 is in charge of the interaction with the nuclear receptor co-repressor 1 binding region of RAR and thyroid receptor (44).

In conclusion, an increasing number of evidence demonstrates the regulation of thyroid hormones by vitamin A and its derivatives from the gene expression level to their effect on target tissues.

## 6 Beta-carotene level in hypothyroidism: past history and molecular mechanism

Hypothyroidism is a prevalent disorder that is more common in women, the elderly, and certain ethnic groups. Clinical hypothyroidism is characterized by high levels of TSH and low FT4 levels. In contrast, subclinical hypothyroidism is characterized by normal FT4 levels and high serum TSH levels. For instance, the hypothalamic, pituitary, and thyroid axis must be unimpaired. There must be no associated disease, and this pattern must be persistent for a minimum of 4 weeks. The subjective nature of the symptoms of hypothyroidism varies with the level of biochemical thyroid hormones. Common symptoms include weariness, cold intolerance, dry skin, constipation, voice changes, and aches and pains in the muscles. The sensitivity and specificity of these symptoms and the scoring system to identify hypothyroidism are inadequate (45). Moreover, patients with overt hypothyroidism express more and more significant symptoms. Hypothyroidism is mainly diagnosed with biochemical changes rather than clinical symptoms (46). Indeed, in adults, the upper limit of the TSH baseline range typically rises with age. Additionally, patients have their TSH reference range, which effectively spans just 25% of the general reference range (47). Currently, the monotherapy of L-thyroxine is the first-line treatment of hypothyroidism to normalize the T3 and T4 levels; also, it is approved that patients with higher TSH levels (≥10 mIU/L) should receive medication. The initial dose of L-thyroxine is 50 mg daily; later, in non-pregnant individuals, experts suggest that the dose of L-thyroxine be adjusted by maintaining the TSH level within the standard reference range (48). Furthermore, recent clinical trials suggest combination therapy of L-thyroxine and LT3, which had better results in preventing disease progression (49, 50).

**Figure 2:**
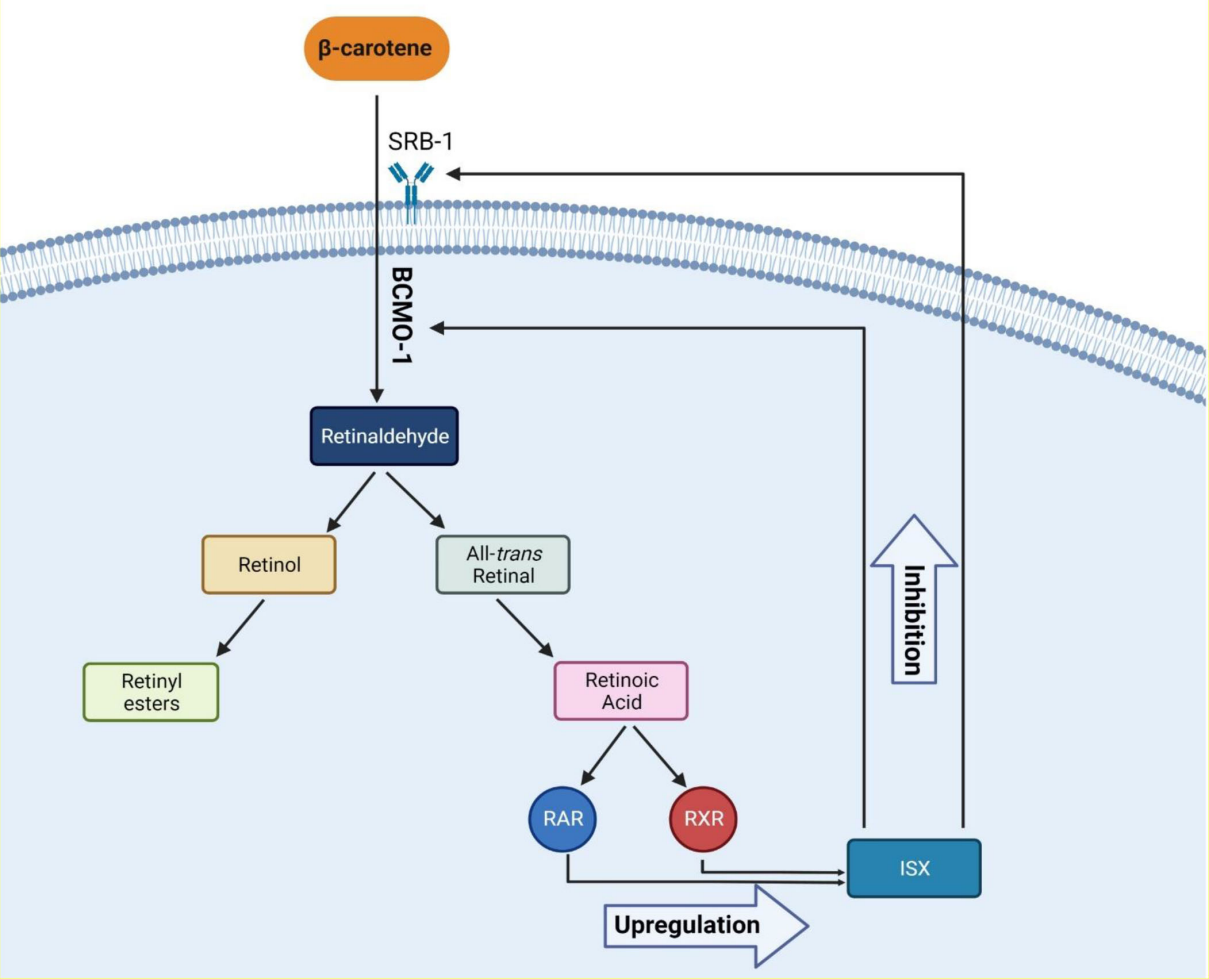
The proposed molecular mechanism of β-carotene metabolism and its effects. Abbreviations: RAR, retinoic acid receptor; RXR, retinoid X receptor; ISX, intestinal transcription Factor; SRB-1, scavenger receptor class B type 1; BCMO-1, beta-carotene oxygenase 1.

Several factors cause hypothyroidism. The most prevalent cause of hypothyroidism is iodine deficiency, one of the crucial elements of thyroid hormones (51). Recently, it was believed that the high profile of β-carotene is also related to hypothyroidism. There is evidence that thyroid hormones are associated with retinol and β-carotene metabolism (52). In a recent trial of 101 patients, Kiuchi et al. reported that high levels of β-carotene were associated with a lower free T4 and a high concentration of TSH in plasma; the prevalence of high serum β-carotene was 8% in patients with primary hypothyroidism and 6.5% in patients with subclinical hypothyroidism (27). Also, in another study, Goswami et al., in agreement with a previous study, reported that in women with hypothyroidism, higher levels of β-carotene and retinol were observed; however, the mentioned study did not include men (53). As a result, it is believed that the high β-carotene level is correlated with a lower level of T4; however, in terms of retinol, there are controversial reports. Aktuna et al. reported that in hypothyroidism, β-carotene levels were substantially greater than in hyperthyroidism. In addition, serum retinol concentrations did not differ between hypothyroidism, euthyroidism, and hyperthyroidism, nor was a substantial rise found in hypothyroidism (52). T3 increases CMO1 (BCMO1) mRNA expression and enzyme performance in Caco-2 BBe cells in the human intestine, resulting in high serum β-carotene profile in patients with hypothyroidism. Furthermore, the conversion of β-carotene to retinol is inhibited in patients with hypothyroidism, and a negative relationship persists between β-carotene and retinol (54). In adults, RAR, RXR heterodimer, intestine-specific homeodomain transcription factor, and intestinal scavenger class B type 1 regulate β-carotene intake and intestinal BCMO1 activity. With sufficient vitamin A, intestinal transcription factor (ISX) inhibits both BCMO1 activity and intestine β-carotene intake *via* SRB1. ISX regulation, which suppresses BCMO1 and SRB1, is decreased in vitamin A deficiency (VAD) due to decreased retinoic acid profile, resulting in normal regulation of BCMO1 and SRB1. One possible explanation for the relationship between β-carotene and THs is that β-carotene is absorbed by cells in the intestine, which may lead to lowering the levels of THs in individuals with β-carotenemia, and this is supported by current evidence. In conclusion, in order to understand the exact relationship of retinol and β-carotene in thyroid disorders, further *in vivo* and *in vitro* studies are required (55). **Figure 2** shows the proposed molecular mechanism of action.

## 7 Beta-carotene level in hyperthyroidism and its possible mechanism of action

The clinical condition known as hyperthyroidism is carried on by the blood rising level of thyroid hormones. In the United States, the overall prevalence of hyperthyroidism is 1.2% compared with 0.5% in overt hyperthyroidism and 0.7% in subclinical hyperthyroidism (56). The most prevalent cause of hyperthyroidism is Graves’ disease. Graves’ disease is an autoimmune condition in which antibodies activate TSH receptors and increase TH secretion (51). Hyperthyroidism can cause a variety of symptoms such as heat intolerance, sweating, trembling, widened eyes, drooping eyelids, weight loss, muscle weakness, and psychiatric conditions (57). Several laboratory tests are used in the diagnosis of hyperthyroidism. Low TSH and elevated levels of free T4 are usually characterized as laboratory findings of hyperthyroidism. Also, we have to mention that free T3 tests are not reliable (58). Furthermore, the radioactive iodine uptake test quantifies the amount of iodine that is absorbed by the thyroid gland. An elevated level of iodine uptake is commonly observed in individuals with Graves’ disease, as opposed to those with a toxic multinodular goiter or an adenoma (59). The treatment options for hyperthyroidism range from conservative therapy to total thyroidectomy. Despite the originating cause of hyperthyroidism, beta-blockers suppress the adrenergic symptoms. Theoretically, propranolol also inhibits 5-monodeiodinase, preventing the peripheral conversion of T4 to T3. The management pathway for hyperthyroidism produced by an excess of thyroid hormones is determined by the patient’s age, symptoms, comorbidities, and individual preferences (60). As previously mentioned, the potential link between hyperthyroidism and elevated β-carotene levels remains unclear. Additional preclinical and clinical studies are needed to fully understand the link between hyperthyroidism and β-carotene. Although numerous biochemical factors accompany hyperthyroidism, an increase in β-carotene or a decrease in vitamin A levels is not among them. Further investigation is required to elucidate the exact correlation between elevated thyroid hormone levels and the presence of β-carotene and retinol.

## 8 Retinoid and the inhibition of TSH secretion

**Figure 3:**
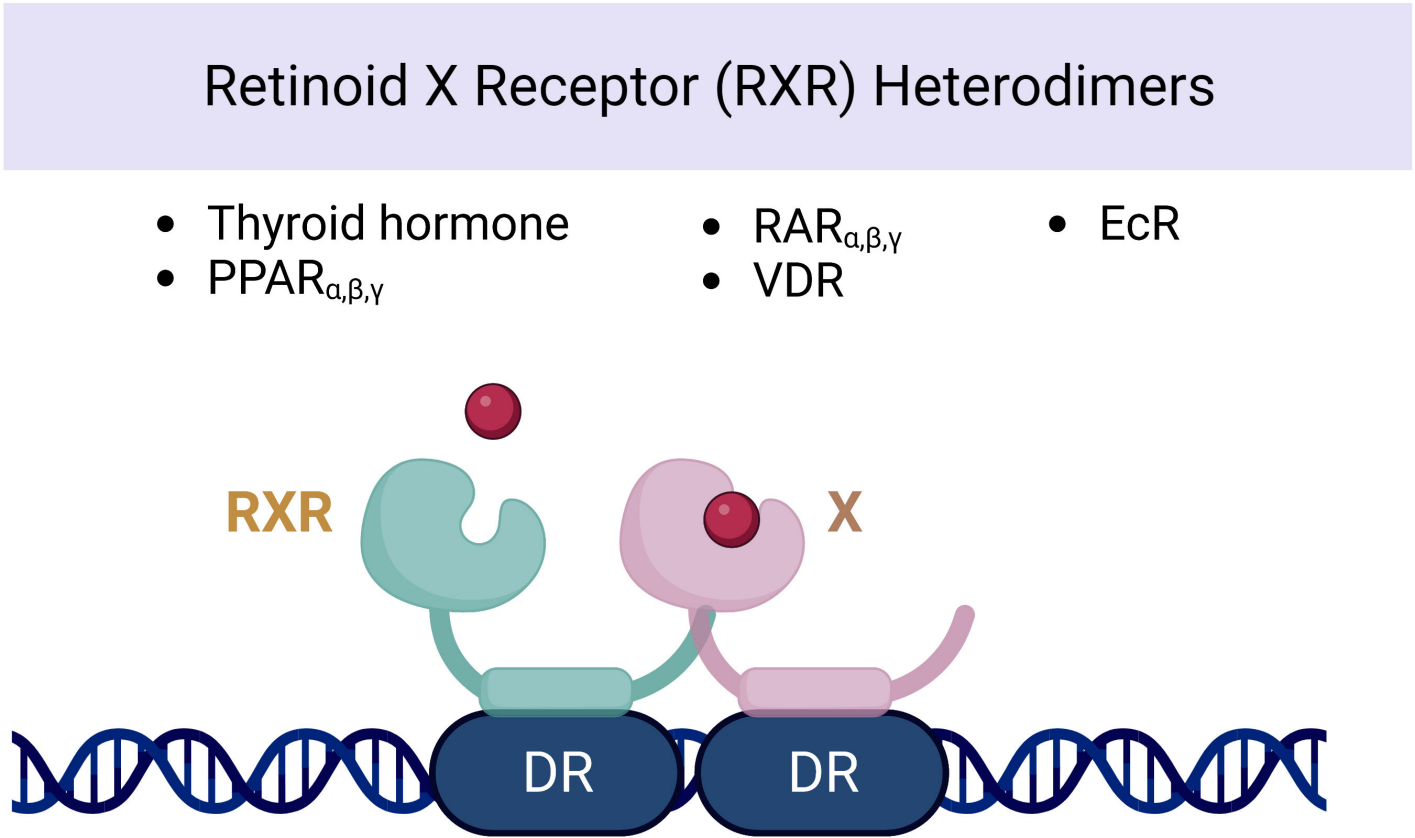
Nuclear receptor of retinoid X heterodimers and its association with thyroid hormonal secretion.

Several observational studies have reported a correlation between VAD and thyroid dysfunction or enlargement of the thyroid gland (goiter) (61, 62). **Figure 3** represents the schematic nuclear receptor of retinoid X heterodimers and its association with thyroid hormonal secretion. Studies in children with severe goiter have shown lower serum retinol and retinol-binding protein levels compared with children with mild or no goiter (63). Various impacts on the pituitary–thyroid axis have been observed in relation to VAD. The level of vitamin A plays a role in regulating both thyroid metabolism (64) and peripheral thyroid hormone metabolism (65–67). An *in vitro* preclinical study on rats demonstrated that vitamin A influences the secretion of thyrotropin or TSH by the pituitary gland. Moreover, VAD leads to enlargement of the thyroid gland (68) and reduced thyroid iodine uptake (69). VAD might also affect thyroid metabolism *via* a central mechanism. In the central axis, the thyroid hormone receptor is found in two isoforms: α and β (70, 71). The effect of RA on the expression of the pituitary TSHβ gene was investigated through its interaction with the RXR receptor. It was found that RA inhibits gene expression by binding to half-sites located on the promoter DNA. However, no significant effect was observed on the hypothalamic TRH level. Such findings suggest a potential mechanism for regulating thyroid hormone synthesis and secretion (72). Additionally, the authors discovered that retinoids increased thyroid hormone metabolic clearance *via* mechanisms mediated by deiodinase and non-deiodinase enzymes in some organs such as the liver and pituitary gland. The administration of VAD in rats increased the levels of plasma T3, T4, and free T4 index, as well as pituitary TSHβ mRNA. Despite the presence of high serum TT4, the expression of elevated TSH mRNA indicates that the impact of VAD has caused a general insensitivity in the pituitary thyrotrope’s response to thyroid hormone. This suggests that further investigation into the mechanisms behind this effect may be warranted. In patients with cutaneous T-cell lymphoma receiving bexarotene therapy (synthetic retinoid), blood TSH levels and free T4 have been found to decline (70). In one report, 19 of 27 patients with bexarotene therapy experienced symptoms of hypothyroidism (fatigue, constipation). High TSH suppression was observed in patients receiving high doses of bexarotene (>300 mg/m^2^ daily) (71). In a double-blind, randomized study of six healthy subjects treated with a single dose of bexarotene (400 mg/m^2^) or placebo, plasma TSH levels decreased as early as 12 h after therapy and reached a nadir at 24 h. Free T4 and free T3 levels were also significantly lower than placebo over 48 h (73).

## 9 Retinoid influences the glycoprotein α-subunit and TRH gene expressions

Pituitary glycoprotein hormones are formed by two different subunits, the alpha (α) and beta (β) subunits. Among FSH, LH, and TSH hormones, the alpha subunit is common, but the beta subunit is unique for each of the three hormones (74). Retinoids are a group of vitamin A derivatives that are part of the vision system (11-cis-retinal), and they regulate the genes involved in cell death: how they differentiate and proliferate. Vitamin A is transformed by enzymes to all-trans-retinoic acid; after that, it is converted to isotretinoin and 9-cis-retinoic acid in the microsomes of the liver (70). Two groups of nuclear receptors mediate the activity of all-trans- and 9-cis-retinoic acid: one is called the RXRs and the other one is RARs (75, 76). Both 9-cis- and all-trans-retinoic acids can activate RARs, but on the other hand, 9-cis-retinoic acid, unsaturated fatty acids, and many natural or synthetic ligands named rexinoids can activate RXRs (77). LGD1069 called bexarotene and LG100268 are two of the highly RXR-selective synthetic rexinoids (78). Janssen et al. in their preclinical study among murine TαT1 thyrotrope cells found that a retinoid X receptor antagonist named LG101208 makes a 71%–81% increase in the levels of TSHβ mRNA *in vitro* at 24 and 48 h and a 47% to 53% increase in the levels of D2 and glycoprotein α-subunit mRNA, and the RXR agonist LG100268 decreases the levels of the glycoprotein α-subunit, TSHβ, and D2 mRNA (79).

Hypothalamus TRH controls TH production. The TRH gene promoter site is made up of three isolated sites that altogether suppress the promoter. Two of the three sites only bind to TH receptor monomers, while the last one can bind to monomers or homodimers of the TH receptor and even to heterodimers of TR/RXR (80). The *in vivo* usage of LG100268 among mice in a preclinical study decreased serum TSH and T4 levels but had no effects on hypothalamic TRH mRNA levels (70). Accordingly, after RXR antagonist usage, similar hypothalamic preproTRH mRNA levels were observed in murines compared with the controls, despite raised T4 levels (79). Altogether, bexarotene is demonstrated to cause central hypothyroidism by affecting the TSHβ gene regulatory regions and concurrent influence on the α-subunit and TRH synthesis (81). Sharma et al. in an experiment in TaT1 cells and after 48 h of treatment with LG100268 (RXR selective) and TTNPB (RAR selective) found that treatment with LG100268 significantly reduced both TSH subunits and D2 mRNA levels, whereas TTNPB showed no influence (70). In summary, retinoid agonists decrease glycoprotein α-subunit and TRH gene expressions, and retinoid antagonists increase them.

## 10 Retinoid in thyroid eye disease

Thyroid eye disease (TED) is an autoimmune infiltrative disease known as the most common cause of orbitopathy or dysthyroid ophthalmopathy worldwide (82, 83), found in up to 50% of patients with Graves’ disease (GD) (84). Graves’ orbitopathy (GO) pathogenesis is uncertain and the underlying cause remains undetected (85). The clinical features of GO consist of proptosis, eyelid edema, diplopia, corneal abrasion, and optic neuropathy (86, 87). GO standard treatment is based on corticosteroids and radiotherapy (RT) to reduce the activity and duration of the disease. Oxidative stress plays a significant role in the worsening of TED leading to the investigation of antioxidants such as selenium, which have the potential to limit the progression of TED (88). Increased oxidative stress is a defining feature of hyperthyroidism (89). It appears that increases in many types of oxidative stress are involved in GO progression (89, 90). Antioxidants have been studied to find out how they counteract the effects of oxidative stress in TED (88, 90–93). This imbalance of antioxidants in TED patients is due to the growth of orbital fibroblasts, synthesis of autoantibodies, degradation of preadipocytes to adipocytes, and secretion of endogenous cytokines (TNFα, IL1-β, and IFNγ), which consequently leads to fibro-adipose tissue development and inflammation of the extraocular muscle. A few studies have tried to find out the role of antioxidants in autoimmune hyperthyroidism and thyroiditis in TED treatment, with conflicting results regarding the effects of different types of antioxidants. Rotondo Dottore et al. reported that retinol, beta-carotene, and vitamin E significantly reduced H_2_O_2_-induced secretion of glutathione disulfide and IL-1 in GD, but not in control fibroblasts. Beta-carotene increased orbital fibroblast growth in GD and retinol decreased IFN-γ in GD and controlled fibroblasts (94). In a systematic review about the effect of antioxidants in TED, results showed that β-carotene, retinol, N-acetyl-l-cysteine (NAC), vitamin C, melatonin, resveratrol, vitamin E, and quercetin may have some efficacy in the management of TED. Although various antioxidants have shown potential for managing TED, there is insufficient evidence to support the implementation of any specific antioxidant or combination thereof in routine clinical practice. It appears that more clinical studies are needed to confirm the effects of beta-carotene on GD (95).

## 11 Beta-carotene thyroid cancer association and its possible mechanism of action

RAs modulate cell division and development of various cell types by binding to a RAR and also RXR (96). Each receptor has three subtypes, namely, α, β, γ, and different analogs of RA have been utilized for the treatment and chemoprophylaxis of neoplasms such as acute promyelocytic leukemia (97) and skin carcinoma (98, 99). Recently, a reduction of RAR-β expression has been discovered in several malignancies, such as non-small cell lung carcinoma, squamous cell carcinoma of the head and neck, and breast and cervical cancers. Some studies show that downregulation of RAR-β and RXR-β correlates with end-stage lung cancer (100, 101). Rochaix et al. (102) reported that reduced RAR-β protein expression was found in all cases of papillary thyroid carcinoma (PTC) and in 50% of cases of follicular carcinoma. Carotenoid concentration is an indicator of PTCs, and underexpression of RA receptors also appears to be related to PTC deterioration and stage, likely providing an additional diagnostic indicator of PTC. Differentiated thyroid carcinomas (DTCs) have a mild proliferation that can be completely treated with the combination therapy of surgery and radioiodine therapy. The efficacy of therapeutic methods is reduced in cases of dedifferentiated thyroid carcinomas. *In vitro* studies have demonstrated the potential of RA therapy to restore thyroid cancer cells to their original specialized state, as evidenced by the increased transcription of thyroid-specific proteins (103–106) and elevated cellular radioiodine uptake (107). RA also plays a role in opposite growth pathways, such as limiting cell division and stimulating cell death. Simon et al. (108) reported an investigation of the efficiency of RA treatment for end-stage thyroid carcinomas (13-cis-retinoic acid: 1.5 mg/kg/day for 5 weeks). A positive effect was observed in 20% of the patients, in whom tumor regression or stabilization correlated with decreased Tg plasma level or increased iodine uptake. Malignancy size showed no alteration or regression in 56% of the patients.

## 12 The relation between vitamin A and thyroid function in pregnant and obese individuals

Thyroid physiologic changes during pregnancy include a moderate increase in gland size and vascularization. Thyroid stimulation occurs since the first trimester, caused by beta-human chorionic gonadotropin (β-HCG), due to structural similarity with TSH (109). β-HCG has a thyrotropic activity that causes a decrease in serum TSH in the first trimester (110). There is estrogen stimulation and the level of circulating thyroid-binding globulin (TBG) in the blood increases (111). In early pregnancy, total thyroxine (TT4) and triiodothyronine (TT3) are increased and peak in the second trimester (112). Generally, pregnant women have lower free-hormone concentrations compared with non-pregnant women (113, 114). During pregnancy, thyroglobulin often rises, indicating increased thyroid activity (115). The fetal THs are synthesized after 12 weeks of gestation; before this time, maternal THs are responsible for the physiological development of the fetal brain (116, 117). Vitamin A is essential for pregnant women to maintain night vision and for fetal ocular health, fetal skeleton development, and immune system maintenance (118–121). The most important adverse effects caused by additional vitamin A intake, especially in the early stages of pregnancy, are spontaneous abortion and congenital anomalies of the central nervous and cardiovascular systems (122, 123). Teratogenicity risk is increased when daily usage of vitamin A exceeds 10,000 IU. Malformations (e.g., urinary tract malformations) are reported in children whom their mothers use high doses of vitamin A (>25,000 IU/day) (124, 125).

The prevalence of obesity is increasing worldwide with lifestyle and environmental alterations (126). Disorders of glucose and lipid and vitamin deficiency are common in obese individuals (127). Obese individuals are vitamin-deficient, particularly the fat-soluble vitamins like vitamin A (VA) (127). VA is significantly lower in individuals with metabolic syndrome (128, 129). Obesity and TH dysfunction are significantly linked (130, 131). Iodine is required for the metabolism of THs as well as micronutrients such as VA (25). Thyroid metabolism, TH synthesis, TH peripheral function, and TSH secretion are all regulated by VA (68, 132). The synthesis of thyroglobulin, T4 and T3 formation, and thyroid iodine uptake are affected by VAD (68, 132), while VA supplementation reduces TSH, mean thyroglobulin, and thyroid size (133). Bingwei Ma et al. in a study found that subclinically hypothyroid (SH) obese had lower VA levels than euthyroid obese and also VA-deficient obese had lower FT4 and higher TSH than normal obese (134). Farhangi et al. in an interventional study found that VA supplementation in both obese and non-obese women significantly decreases TSH and increases serum T3 (34). This might be due to decreased responsiveness of tissue to THs or increased hepatic conversion of T4 to T3 by VA supplementation as reported by Morley et al. (135). In summary, obesity is associated with lower vitamin A and elevated TSH levels, and vitamin A supplementation might reduce the risk of hypothyroidism.

## 13 β-carotene conversion products and their effects on thyroid hormone level

**Figure 4:**
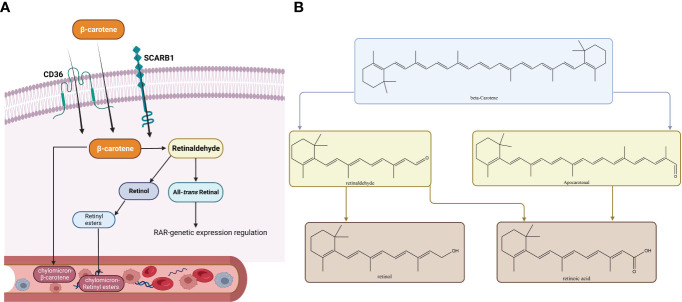
Metabolic pathways of β-carotene. The membrane proteins SCARB1 and CD36 can mediate absorption in the intestinal epithelium, or absorption can occur passively by diffusion. When inside the cytoplasm of an enterocyte, there are two ways a substance can be metabolized. **(A)** The metabolic route most frequently taken, which culminates in the release of retinyl esters (RE) or β-carotene into the bloodstream in tandem with chylomicrons. **(B)** Both the conventional metabolic route and the alternate cleavage-generating apo-carotenal molecule are depicted. Similar metabolic by-products are produced by both substances (all-trans-RA, ATRA). Abbreviations: RAL, retinal; ROH, retinol; B1ChM-β, carotene–chylomicron–β-carotene; CD36, cluster of differentiation 36; SCARB1, scavenger receptor class; ChM-RE, chylomicron-RE.

All the fruits that are colored and leafy vegetables that are green have pigments called carotenoids (136). Carotenoids are various, and among them, β-carotene has provitamin A activity and is assumed to be of great nutritional value. Up to 80% of our daily vitamin A originates from provitamin A carotenoids which can be found in fruits and vegetables (137, 138). Animals cannot synthesize vitamin A. It is supplied as animal food resources or as provitamin A carotenoids (139). Vitamin A, which is called retinol, and RA as its active metabolite play key roles in the growth and differentiation of cells and vertebral development (139). Vitamin A deficiency may lead to xerophthalmia, immune system impairment, blindness, and a higher risk of death (137). β‐Carotene supplementation can fight against vitamin A deficiency complications such as reducing night blindness incidence (140–143). Absorbed β-carotene is metabolized throughout centric and eccentric pathways. The centric pathway is carried out by β-carotene 15,15'-monooxygenase enzyme (BCO1) which produces retinal, and in the eccentric pathway, apo-10'-carotenal is produced by β-carotene 9',10'-dioxygenase enzyme (BCO2). Retinal dehydrogenase enzymes metabolize retinal to retinol, and after esterification to retinyl esters, it is packed with non-converted β-carotene into chylomicrons (144, 145). Toxicity is not observed in individuals consuming large amounts of β-carotene because of the reduction in vitamin A production efficiency at increasing doses (137, 146, 147). Vitamin A alterations on thyroid function include alteration in the TH-binding capacity of the serum proteins, decrease in total T4 and T3 levels, increase in thyroid radioactive iodine uptake, and alteration in T4 to T3 conversion (135). McCarrison in a preclinical study found that a deficiency of vitamin A leads to rat thyroid hypertrophy (148). Drill found that vitamin A deficiency leads to hypertrophy of the thyroid gland and excessive vitamin A decreases the amount of thyroid colloid (149). Morley et al. found an integral thyroxine and vitamin A relationship. An increase in vitamin A leads to TH reduction, and vitamin A deficiency leads to an increase in THs (150). Di Bella et al., in a preclinical study among rats, demonstrated that vitamin A affects the thyroid gland by producing a dwindled gland, higher radioiodine uptake, and enhanced hepatic conversion of T4 to T3. Thyroid gland overactivity may be explained by vitamin A-based stimulation of cathepsin activity which controls proteolysis (151). In summary, β-carotene in the human body converts to vitamin A which if decreased leads to thyroid hypertrophy and TH increase. Higher vitamin A intake may increase radioiodine uptake and raise the hepatic conversion of T4 to T3. In addition, the β-carotene-metabolizing pathways are shown in **Figure 4**.

## 14 The effect of hypo/hyperthyroidism and their association with antioxidant status

Oxygen-free radicals have an effect on the mechanism of tissue damage of multiple pathologic pathways (152), including hypothyroidism and hyperthyroidism (153–155). The accessible data related to oxidant stress and antioxidant role in hypothyroidism are rare and inconsistent. Hypothyroidism is a decrease in free radical production as a result of the metabolic suppression brought about by the reduction in serum thyroid level (154, 156). However, other lines of evidence reported that hypothyroidism increased oxidative stress (157), and hyperthyroidism is associated with a metabolic increase and, therefore, an increased amount of oxidative stress and peroxides and even an increase (158–160) or a decrease (161) in antioxidant enzymes. Studies showed that hyperthyroidism treatment reduced oxidative stress (162). A study examined the effects of hyperthyroidism on the antioxidant defense system, specifically serum levels of thiobarbituric acid-reactive substance (TBARS), vitamin E, and coenzyme Q10. The results show that hyperthyroidism leads to increased TBARS and reduced antioxidants. However, these values were normalized in euthyroid patients. In addition, studies take into account the possible negative effects of hyperthyroidism on important antioxidant enzymes such as Mn or Cu, Zn, superoxide dismutase, catalase, and glutathione peroxidase (163). In the systemic investigation, hyperthyroidism had a role in the decline of serum alpha-tocopherol (162, 164) and CoQ10 in humans (162, 165). Baskow et al. found in hypothyroid patients (166) a high malondialdehyde (MDA) level, an oxidative stress agent that is made by lipid peroxidation, as well as NO, and a low activity of paraoxonase-1 (PON-1), a liver enzyme made with an antioxidant agent. On the other hand, levothyroxine therapy reduced MDA levels and increased PON-1 activity, although several similar studies had different conclusions (166). They claimed that in hypothyroidism the prooxidant environment could affect the development of atherosclerosis. MDA rising levels also represent mild thyroid failure (167). Increased OS has a role in the reduction of antioxidants and also leads to hyperlipidemia. Furthermore, a considerable association exists between MDA and LDL-cholesterol, serum cholesterol, and triglyceride levels. The antioxidant level was not significantly different in clinical hypothyroidism, mild thyroid failure, and controls. OS can also damage the thyroid itself, which appears in iodine-excess conditions. This matter was investigated both *in vitro* and in animals (168, 169). Iodide induced action on hydrogen peroxide production in the thyroid and damaged thyrocytes at high accumulation (170). Subudhi and colleagues discovered that when hypothyroid rats were given vitamin E and curcumin supplements, the mitochondrial oxidative stress levels of their livers were improved and hyperlipidemia was reduced (171). **Table 2** is a summary of reports on β-carotene and vitamin A levels in association with thyroid hormones.

**Table 2:** Reports of β-carotene and vitamin A levels in association with thyroid hormones.

Author	Year	Status	Outcome	Ref
Sachiko Kiuchi et al.	2018	β-Carotene and hypothyroidism	The prevalence of hyper β-carotenemia in patients with primary hypothyroidism is 8%, and in patients with subclinical hypothyroidism, the prevalence is 6.5%.	(27)
Goswami UC et al.	1999	β-Carotene, hypothyroidism, and hyperthyroidism	β-Carotene and retinol levels were elevated in hypothyroidism but were decreased in hyperthyroidism.	(53)
Steven I. Sherman et al.	1999	Retinoid and thyroid hormones in a patient with cutaneous T-cell lymphoma	Retinoid treatment of the patient with cutaneous T-cell lymphoma decreases the level of thyroid hormones.	(71)
A. R. Angioni et al.	2005	Retinoid and TSH	Chronic retinoid intake reduces the level of TSH in serum.	(172)
Wendy M. Golden et al.	2007	Retinoid and TSH	Retinoid administration can reduce TSH concertation in healthy adults.	(73)
Mervat M. El-Eshmawy et al.	2016	Vitamin A and thyroid hormones in patients with hepatitis C virus	Vitamin A deficiency is associated with hyperthyroidism condition.	(173)
Mahdieh Abbasalizad Farhangi et al.	2012	Vitamin A and TSH in premenopausal women	Vitamin A treatment may decrease the likelihood of subclinical hypothyroidism in premenopausal women since serum TSH levels were considerably reduced in vitamin A-treated participants.	(34)
G. Ceresini et al.	2002	Vitamin A and TSH	In healthy individuals, treatment of vitamin A appears to have little effect on TSH production; the findings also imply that the interaction between T3 and vitamin A in the regulation of TSH secretion is unlikely.	(174)
RK Miller et al.	1998	Safe dosage of vitamin A and β-carotene in pregnant women	It is believed that a daily dose of 10,000 IU/day is safe in pregnant women, and intake of β-carotene is not teratogenic at any dose.	(122)

## 15 Recent clinical studies

Due to the importance of the thyroid gland in maintaining normal metabolism in the human body, many researchers have tried to evaluate the crosstalk between vitamin A derivatives and THs in human subjects and compare the results of animal studies at a clinical level. In developing countries, because of the prevalence of iodine and vitamin A co-deficiency, the risk of thyroid disorders, particularly in children, has been of interest. Zimmermann et al., in a study on 404 children from regions with mild to moderate iodine and vitamin A deficiency, showed that vitamin A supplementation caused a significant decrease in mean thyroglobulin, median TSH, and thyroid volume. They proposed that in the presence of vitamin A, the thyroid gland is less triggered by excess TSH and the chance of goiter is reduced (133). In a similar study of 138 pediatric patients with goiter, vitamin A supplementation in combination with iodized salt reduced thyroid volume and serum TSH levels more significantly than iodized salt alone (36). Similar studies have also been conducted on adult populations. The studies mainly focused on assessing the impact of vitamin A supplementation on the thyroid function of patients with thyroid disorders. However, some researchers evaluated the baseline status of vitamin A in patients with such diseases. For instance, Ibrahim et al. studied the nutritional condition of female patients with thyroid disorders. They found that in women with hyperthyroidism, the average vitamin A intake was considerably higher than in those with hypothyroidism (175). Farhangi et al. in a randomized controlled trial assessed the effects of vitamin A supplementation on thyroid status in obese women. The obese women received placebo or vitamin and non-obese subjects received vitamin A. They found that vitamin A supplementation led to a noticeable decrease in TSH levels in both obese and non-obese subjects. Furthermore, serum T3 levels rose in both treated groups, while serum T4 levels dropped in the three groups after the trial. Their findings confirmed a marked decrease in serum retinol-binding protein in the obese group treated with vitamin A, but no meaningful change was detected in serum transthyretin (34). Haugen reported that a high dose of vitamin A was effective in reducing symptoms and metabolic rate in individuals suffering from hyperthyroidism (35). On the other hand, recent studies have shown the benefits of vitamin A supplementation in individuals with hypothyroidism. As an illustration, Rabbani et al. designed a randomized controlled trial where 86 hypothyroid patients either were treated with supplementation including zinc gluconate, magnesium oxide, and vitamin A or received placebo. They demonstrated that in the intervention group, serum free T4 concentrations elevated considerably, while after the intervention, the differences in serum total T4, TSH, and free T3 were not significant (33). As might be noticed, the results of previous studies in terms of the effects of vitamin A on THs have been controversial. These discrepancies might be justified by the presence of complex feedback between the hypothalamus–pituitary–thyroid axis (176). In T4, as most of this hormone is bound to transporter proteins, the alterations in its serum levels may be masked. Regarding TSH, T3 is the main regulator of TSH release from the pituitary gland negatively influencing TSH synthesis. Thus, a lack of alteration in serum FT3 can lead to no change in TSH levels (33). Overall, despite differences between the findings of previous studies on this topic, there is a strong body of evidence on the potential of vitamin A supplementation in the treatment of thyroid disorders in human subjects.

## 16 Discussion and future perspectives

**Figure 5:**
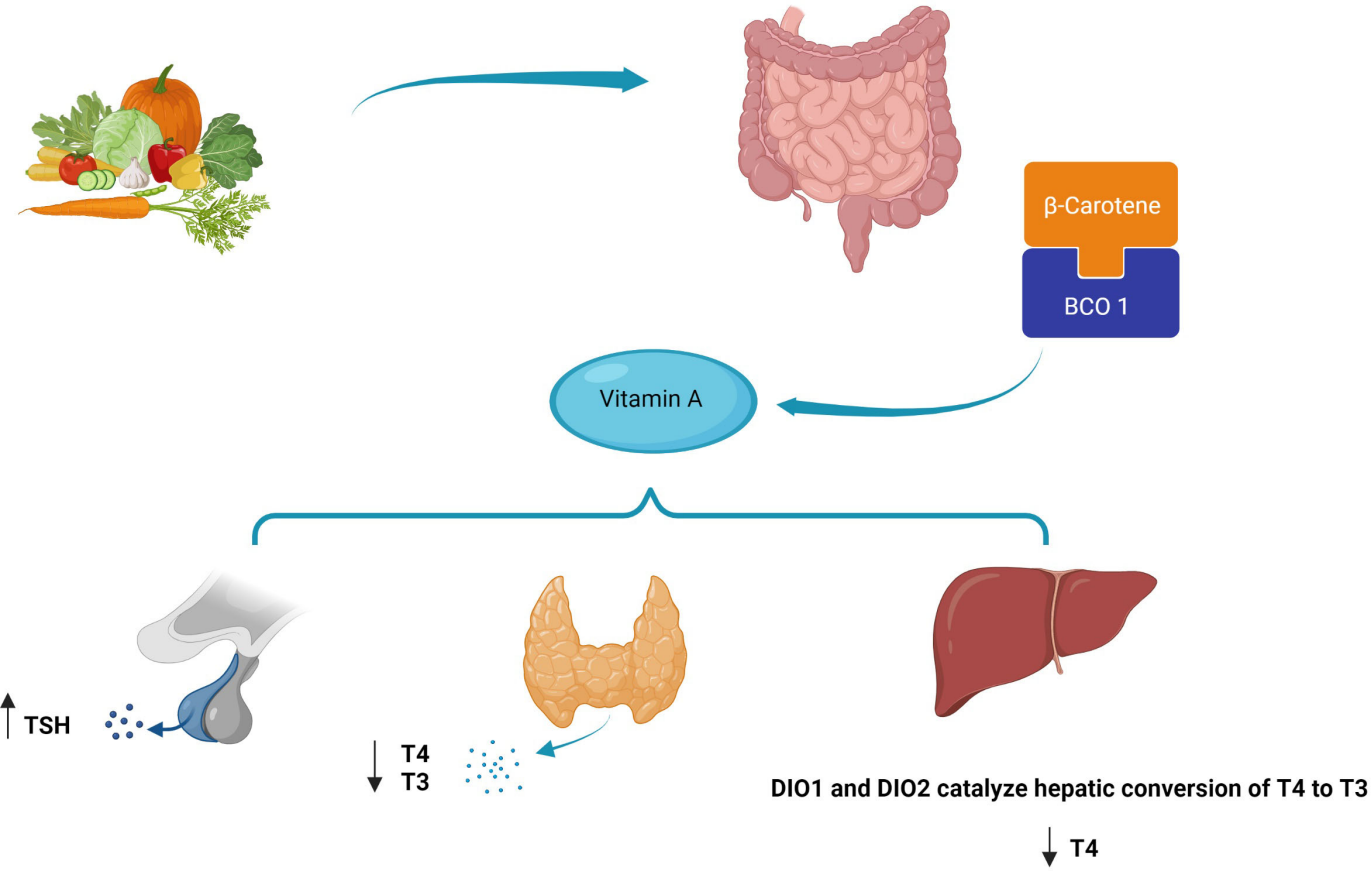
Proposed mechanism of beta-carotene’s role in affecting thyroid hormones.

TH receptors are nuclear receptors that require heterodimerization with RXR. In fact, RXR regulates the function of thyroid receptors with the outcome being determined by the ligands of the two receptors (177). Various studies have confirmed the influential role of vitamin A on THs from gene expression to impact on the target cells; a reciprocal effect has also been discovered suggesting the effects of THs on vitamin A metabolism. Hence, vitamin A and thyroid functions are inseparably linked; however, some underlying mechanisms are still unclear (19, 178, 179). In the case of these mutual interactions, a major factor that should be noticed is the accessibility of vitamin A, which is dependent on the equilibrium between the enzymes which are responsible for the production or inactivation of the RA RAR or could be limiting in rigorous nutritional deficiencies (180). This matter becomes more complex knowing the severe impact of vitamin A and iodine co-deficiency on thyroid function in individuals from regions with nutritional crises. Indeed, providing sufficient intake of these micronutrients is a major health issue that needs to be tackled in developing countries (22). Even with providing adequate nutritional intake, there are still complexities in the case of RA–thyroid interactions. RA synthesis and deactivation are regulated *via* complex pathways. Knowledge of the mechanisms can facilitate understanding of the links between RA and endocrine hormones including THs (181, 182). At the clinical levels, the results of studies assessing the impact of vitamin A supplementation on human subjects with thyroid disorders have been controversial yet mostly promising. Recently, the use of vitamin A derivatives as a potential therapeutic option in thyroid cancer has gained the interest of researchers like never before (38, 183, 184). At this moment, we might have effective techniques to tackle some problems in this area. With recent improvements in genome engineering, we will be able to assess the effects of small modifications to RARs/RXRs encoding genes on thyroid hormonal pathways. On larger scales, these types of studies might help discover new pathways in the regulation of the hypothalamus–pituitary axis affecting other endocrine organs. With advances in molecular pharmaceutics and nanoformulation, synergistic drug combinations and polymer micelles might be offered to improve the efficacy of vitamin A derivatives and reduce their adverse effects. Novel machine learning techniques can help analyze large data sets produced by genomic studies and facilitate the understanding of the dynamic associations between RA-related genes and subsequent hormonal processes (185). The proposed mechanism of the role of beta-carotene in affecting thyroid hormones is shown in **Figure 5**.

## 17 Conclusion

In recent years, significant progress has been made in advancing our comprehension of retinoic acid and thyroid interactions at both the molecular and clinical levels. Although the positive effects of vitamin A on patients with thyroid disorders and malignancies are widely accepted, the underlying mechanisms still require further elucidation. Additionally, the efficacy of vitamin A supplementation in thyroid disorders can be improved by conducting large-scale studies utilizing novel pharmacological techniques. These studies will allow for a more comprehensive understanding of the intricate interactions between retinoic acid and thyroid hormones, paving the way for the development of more effective therapeutic strategies. Thus, continued research in this area is crucial for improving patient outcomes and advancing our knowledge of the complex relationship between retinoic acid and thyroid function.

## Author contributions

Data collection: BF, HG and NL. Figures: BF and HS. Review and editing: BF, HG, GM, HS, NL and AR. All authors contributed to the article and approved the submitted version.
